# The unsuitability of html-based colour charts for estimating animal colours – a comment on Berggren and Merilä (2004)

**DOI:** 10.1186/1742-9994-2-14

**Published:** 2005-08-30

**Authors:** Martin Stevens, Innes C Cuthill

**Affiliations:** 1 School of Biological Sciences, University of Bristol, Woodland Road, Bristol, BS8 1UG, UK

## Abstract

**Background:**

A variety of techniques are used to study the colours of animal signals, including the use of visual matching to colour charts. This paper aims to highlight why they are generally an unsatisfactory tool for the measurement and classification of animal colours and why colour codes based on HTML (really RGB) standards, as advocated in a recent paper, are particularly inappropriate. There are many theoretical arguments against the use of colour charts, not least that human colour vision differs markedly from that of most other animals. However, the focus of this paper is the concern that, even when applied to humans, there is no simple 1:1 mapping from an RGB colour space to the perceived colours in a chart (the results are both printer- and illumination-dependent). We support our criticisms with data from colour matching experiments with humans, involving self-made, printed colour charts.

**Results:**

Colour matching experiments with printed charts involving 11 subjects showed that the choices made by individuals were significantly different between charts that had exactly the same RGB values, but were produced from different printers. Furthermore, individual matches tended to vary under different lighting conditions. Spectrophotometry of the colour charts showed that the reflectance spectra of the charts varied greatly between printers and that equal steps in RGB space were often far from equal in terms of reflectance on the printed charts.

**Conclusion:**

In addition to outlining theoretical criticisms of the use of colour charts, our empirical results show that: individuals vary in their perception of colours, that different printers produce strikingly different results when reproducing what should be the same chart, and that the characteristics of the light irradiating the surface do affect colour perception. Therefore, we urge great caution in the use of colour charts to study animal colour signals. They should be used only as a last resort and in full knowledge of their limitations, with specially produced charts made to high industry standards.

## Background

The use of colour charts to estimate or categorise the colours of animal signals is a technique utilised in numerous studies [e.g. [1-5]]. In particular, a recent proposal [1] argues that researchers could produce custom-made charts, designed from the HTML colour code (the standard for colour representation on the World Wide Web). In this paper we present theory and data showing why the use of such charts to estimate colour is seriously flawed and only should be undertaken as a last resort.

### 'Colour is in the eye of the beholder'

A major problem with using colour charts is one frequently stressed: that human vision differs markedly from most animals other than Old World primates [6-11]. Signals are often aimed at specific animals, and it has long been realised that there is an association between the evolution of a particular signal and the receivers' visual system [12], and so signals should be considered from the perspective of the signal receivers' sensory experience [6,13]. The description of a certain colour is something specific to a particular visual system, and this perception may differ greatly between animals [6-8,14].

Colour perception is the product of reflectance, the irradiant light characteristics, the transmission characteristics of the medium, and the characteristics of the animal's visual system [6]. Most of the hues an animal can perceive can be produced by mixing wavelengths of light (called primaries) in different proportions, and so: (a) different light spectra can produce a sensation of the same hue if the output of the animal's photoreceptor types is the same (metamerism), (b) the same spectra will produce different hues to animals that differ in the absorption spectra of their photoreceptors, and (c) the dimensionality of colour space is determined by the number of interacting receptor types [8,15,16]. For instance, birds typically have four single cone types, compared to three in humans, and unlike humans, most birds are capable of perceiving light into the ultraviolet spectrum [[17-24], reviewed by [25-28]]. This means that birds should be capable of perceiving a wider range of hues, and will differ from humans in the magnitude of perceived colour differences, even for those spectra visible to humans.

Whilst avian vision has been described to illustrate why human colour-matching can never quantify the colours perceived by other animals, it is equally important to realise that colour matching to charts of the type proposed in [1] is not even adequate for human perception.

### The inadequacy of colour charts to classify colour

To understand why using certain colour charts to study visual signals is often inadequate, it is helpful to briefly consider some of the main aspects of the colour spaces from which charts are created.

One way to represent colour is to agree on a set of primaries and describe a colour by the values of the weights of those primaries used by subjects to match a test light (additive colour mixing) [29,30]. The CIE (Commission International d'Éclairage) XYZ colour space is one such specification of colour stimuli, produced by additive mixing of three imaginary primaries.

It is usually important to know if a colour difference is perceivable, determined by experimentally modifying colours in small degrees to determine threshold perceptible differences. When plotted in colour space, these differences form the boundary of a region of colours that are indistinguishable from other colours, with ellipses fitted to the boundaries [29]. In colour spaces such as CIE XYZ, the shape and size of the ellipses depends strongly on the location of the difference in the colour space, meaning that the magnitude of the difference in CIE XYZ space is a poor indicator of the real perceived difference between colours [29-31]. Therefore, what is often preferable is a uniform colour space where the distance in coordinate space is comparable to the perceived difference in colour by an observer.

Currently, the most popular uniform colour space is the CIELAB space, obtained by a non-linear transformation of the XYZ space. This colour space is uniform, meaning that equations allow the Euclidian distance between two points in the CIELAB space to predict more accurately the observed difference in colour, although comparisons of 'colour constancy' in CIELAB to empirically measured colour constancy are still often quite poor [32].

It is a misconception that RGB (red, green, blue) colour space is an accurate method to classify colours as seen by humans; it is not (and certainly is not for non-human animals). Indeed, it is not generally associated with studies aiming to match colours to charts. RGB space is most readily associated with colour reproduction on computers, and with its associated CMY colour space for printing. RGB values are those used to represent digital photographs on a colour monitor, with values of R, G, and B usually ranging from 0 to 255 (an 8-bit scale). HTML colour-coding (as advocated by [1]) is simply a concise encoding of the RGB colour format.

There are several criticisms of using RGB colour codes to specify colours from charts. Firstly, RGB space is non-uniform, and therefore differences in RGB values do not equate to equal differences in colour perception. Secondly, unlike the CIELAB space, RGB ratios are not capable of producing all the possible perceptual combinations of colours to humans (let alone to other species). For instance, values of L* = 100, a* = -80 and b* = -2 changing continuously to values of L* = 100, a* = -80 and b* = -59 in CIELAB space, are all represented by the same RGB values (R = 0, G = 255, B = 255), and therefore, differences in the colours produced by CIELAB space over this range simply cannot be reproduced on a computer screen or on a printed chart. Thirdly, and perhaps most importantly, unlike the various CIE colour spaces, the colours generated from RGB colour co-ordinates are device-dependent [31]. That is, a photographic image of a given colour may be represented by different RGB values in different cameras, and a given RGB coordinate in a camera or computer may translate to different colours on different printers.

The experiments in this paper were designed to demonstrate that the faults with Berggren & Merilä's [1] approach are not simply theoretical abstractions. The experiments illustrate the contentions that individual human observers vary in their assessment and ranking of colours, that the surface irradiance characteristics may affect perceptions of colour and, finally, that different printers will produce colour sheets with significantly different reflectance values, even if the RGB/HTML values of the colour charts on a computer are identical (discounting variation arising from different toner levels which are, nonetheless, an important consideration in practice). These are not new arguments, and more thorough experiments are routine in colour science, but rather are presented here to illustrate the pitfalls for those studying animal colouration.

## Results

### Spectrophotometry Results

**Figure 1:**
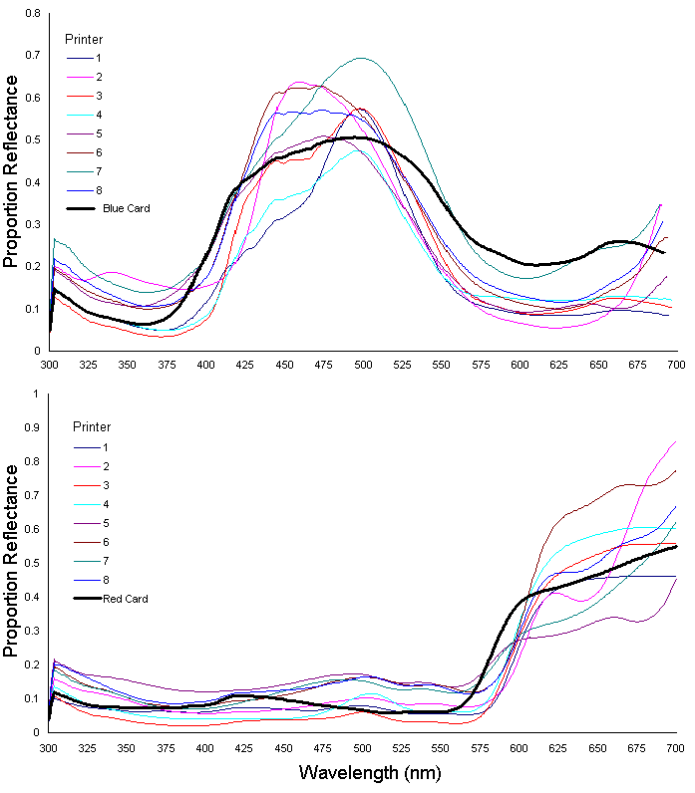
**Spectrophotometry Results Showing the Variation that Exists Between the Different Printers. **Two plots of the average reflectance spectra from the 8 different printer types, for the blue-green colour blocks with B & G values of 200 and R value of 0 (top chart), and the red colour blocks with an R value of 175 and B & G values of 0 (bottom chart). This shows the amount of variation that exists between the different printers, in producing what should have been colour blocks with the same spectra. The spectra of the coloured paint cards used in the matching experiments are included for reference.

**Figure 2:**
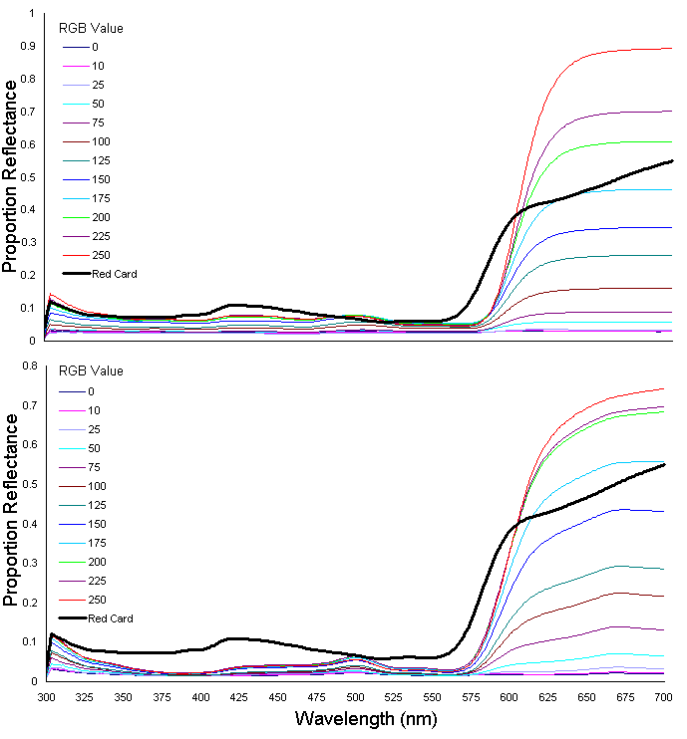
**Spectrophotometry Results Showing the Uneven Scaling of Spectra with Equally Spaced RGB Values. **The two plots show the average reflectance spectra for all the colour blocks included in the study on the red charts, for two of the printers used in the experiments. The top chart shows a 'good' printer, which produced a printed chart with approximately even spacing between the blocks with increasing R-values. This contrasts strongly with the bottom chart, produced from a different printer, which shows an uneven spacing between the spectra, and a 'bunching' of spectra at low and high R-values, meaning that the even spaces in RGB space did not correspond to an even spacing in spectral intensity.

Spectrophotometric data supported the results obtained from the colour matching experiments (below). Reflectance spectra of the colour charts showed that there is significant variation in reflectance (shape and intensity) between the different printer types (Fig. 1). This variation leads to the large diversity in the perceived colours and brightness of the different chart components between printers. No two printers were the same in their reflectance for each of the colour blocks, and this was the case for both the red and the blue-green charts. Also, and perhaps most worryingly, whilst some printers showed a fairly constant increase in reflectance as the R or G/B value increased, many printers produced charts where there were sudden large jumps between what should have been equal steps between the colour blocks, or had several colour blocks at the top end of the RGB values having very similar reflectance (i.e. even parts of the colour chart that were separated by RGB values of 25, 50, or more, would sometimes produce similar reflectance spectra) (Fig. 2), showing that some printers are constrained to smaller variations in colour spacing (lower colour resolution).

### Colour Matching Experiments

**Figure 3:**
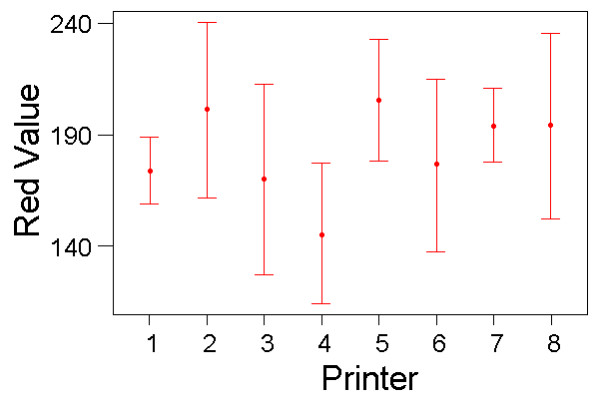
**Colour Matching Results with the Red Charts for the Different Printer Types. **Variation in colour matching choices that subjects made (mean red value on RGB scale, with standard deviation bars) for the eight different red colour charts produced from different printers. Results from all colour matches made, averaged across lighting conditions and subjects.

**Figure 4:**
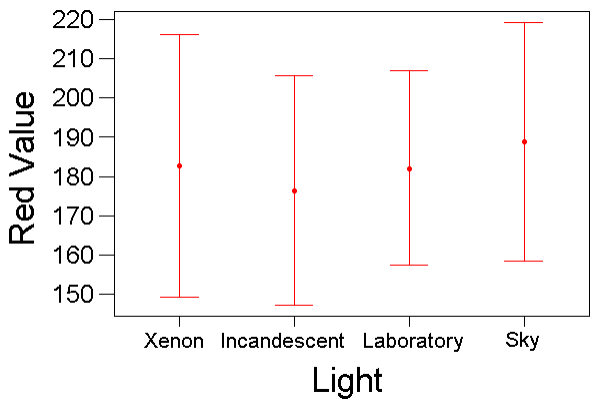
**Colour Matching Results with the Red Charts Under Different Light Conditions. **Variation in colour matching choices that subjects made (mean red value on RGB scale, with standard deviation bars) under the Xenon, incandescent, laboratory and skylight light conditions. Results from all colour matches made, averaged across printers and subjects.

Results from the colour matching experiments showed that for the red charts, there were significant and large effects on subjects' colour matching of the printer and a marginal, non-significant effect of lighting conditions (repeated measures GLM; Printer: F_(7,70) _= 33.00, P < 10^-19^, partial eta^2 ^= 0.767; Light source: F_(3,30) _= 2.74, P = 0.061, partial eta^2 ^= 0.215; Printer*Light source: F_(21,210) _= 1.178, P = 0.273, partial eta^2 ^= 0.105) (Figs 3 &4).

**Figure 5:**
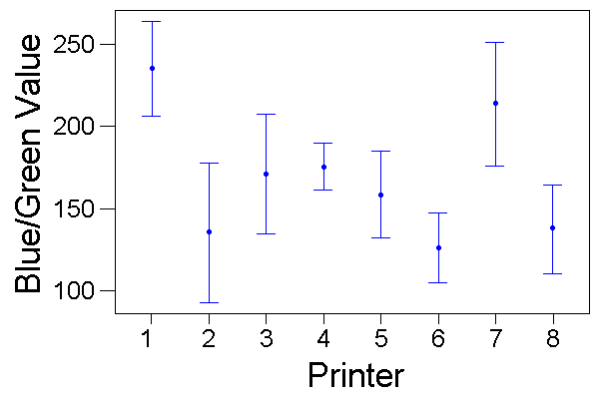
**Colour Matching Results with the Blue-Green Charts for the Different Printer Types. **Variation in colour matching choices that subjects made (mean blue/green value on RGB scale, with standard deviation bars) for the eight different blue-green colour charts produced from different printers. Results from all colour matches made, averaged across lighting conditions and subjects.

**Figure 6:**
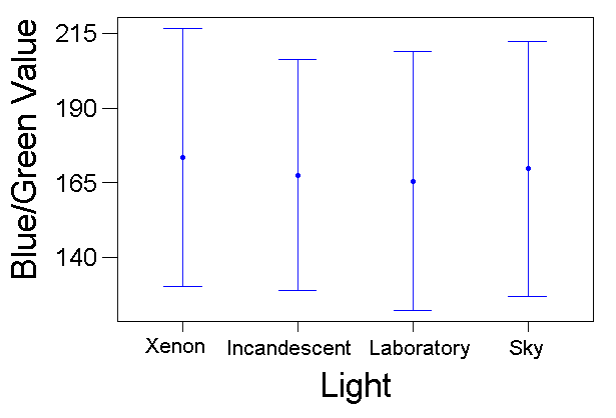
**Colour Matching Results with the Blue-Green Charts Under Different Light Conditions. **Variation in colour matching choices that subjects made (mean blue/green value on RGB scale, with standard deviation bars) under the Xenon, incandescent, laboratory and skylight light conditions. Results from all colour matches made, averaged across printers and subjects.

For the blue-green charts, there was also a significant effect on colour matching of printer (F_(7,70) _= 145.54, P < 10^-38^), with the effect size even greater (partial eta^2 ^= 0.936). Light type had no detectable main effect (F_(3,30) _= 1.40, P = 0.263, partial eta^2 ^= 0.122) but there was a significant light*printer interaction (F_(21,210) _= 2.09, P = 0.005, partial eta^2 ^= 0.173) (Figs 5 &6). The results contained a single large outlier in the data, with a large standardised residual value. However, there was little change to the results of the GLM when this outlier was removed (to determine its potential influence on the data).

These results show that, for both the blue-green and red chart experiments, the colour matching choices that subjects made were very different for charts produced from different printers. There was also a suggestion of a smaller effect of light conditions on colour matching. The variation in colour-matching judgements was large: for the red experiment, the same target colour was matched to chart elements with R-pixel values ranging from 125 to 250 (C.V. = 16%); for the blue experiment, the best match ranged from B/G-pixel values of 75 to 250 (C.V. = 25%). Although not usually interpretable in repeated-measures ANOVA, one could argue that the between-subject variation is of direct interest in this application, because in many applications of colour-matching, only one or a few researchers would be responsible. When treated as a fixed effect, 'subject' was significant for both colour-matching tasks (red: F_(10,210) _= 7.70, P < 10^-9^; blue: F_(10,210) _= 2.55, P = 0.006), although the effect sizes were not as substantial as that of printer type (partial eta^2 ^= 0.268 and 0.108, respectively).

## Discussion

The experiments detailed in this study show three important results with respect to the use of printed colour charts to identify the colours of animal signals. Firstly, people vary with respect to the choices they make when asked to match one colour to the perceived closest match from a set of colours on a chart. This means that there may be differences in colour matches made by different individuals. These differences may, to some extent, be reduced by using high-quality charts with a greater range of colour matching options. Secondly, there were very large and significant differences in the matching choices made by individuals between charts produced from different printers. Different inks have significantly different spectral properties [30]. This is a critical problem of 'self-made' charts, such as those made from RGB colour space. The aim of matching a colour signal to a specific section of a colour chart is that the chromatic content of the signal can be recorded, such as in terms of an RGB value. However, when charts with identical RGB values are produced from different printers these do not have the same properties, making comparisons to an RGB value irrelevant. Even if charts are printed from the same printer, with the same cartridge model and paper type, the exact values of the printed charts will still vary depending upon the toner levels (Cuthill & Stevens unpublished data). This means that complex printer calibrations are needed to ensure that the reproduction of more than one colour chart is accurate and invariable with respect to the chart properties. Furthermore, there still remains the problem that a linear increase in a colour value on a chart may not be linear in terms of the measured reflectance spectra and the perceived difference in colour, for many, if not all colour spaces. Thirdly, and perhaps least expected, there was the suggestion that colour matching results were significantly affected by the irradiant light conditions (non-significant for red charts, at P = 0.06, but in a significant interaction with printer type for the blue chart). For the red charts, the largest mean difference for judgements measured under different light sources was a difference of 14 pixel values on an 8-bit scale. For the blue-green charts, the effect of light varied between charts from different printers: for one printer the average difference in pixel values of best matches was 29, for another it was only 5. That the effects of lighting were modest was expected because humans possess colour constancy, where the visual system is capable of maintaining a constant appearance of colour quasi-independently of changes in the irradiant light. Presumably many other animals also show colour constancy [33-37]. However, the adapting mechanisms are not perfect [38-40] and changes in the irradiant light do have some effect on colour constancy. Different environments can vary significantly in their irradiant light characteristics [41], and thus influence colour appearance.

Whilst in a natural situation, the perception of colours under different conditions will be about the same (i.e. a light red signal will always look light red), in terms of quantitative, or even qualitative scientific experiments, changes in perception may significantly impact upon results, sometimes by large degrees. Results in the field will be affected by the time of day, the weather, and the natural environment [6,41], and will also differ under various laboratory lighting conditions. Finally, charts based on RGB colour space, even if used in studies of human vision, are incapable of reproducing the full range of colours perceptible to humans. The use of charts based on CIE data to estimate colours are better than the use of charts based on RGB colour space, but still unfortunately are based upon human subjective assessment.

The spectrophotometry analysis further supports the argument that printed charts could produce seriously inaccurate colour matching results. Firstly, different printers vary significantly in the reflectance properties of the colour charts that they produce. This means that comparisons between different printers will be unreliable, even discounting the effects of toner level. More seriously however, is the result that some printers do not show even gradations in reflectance between colour blocks with an even spacing in RGB colour space. Therefore, comparing the colours of animal signals between individuals (for example) via charts to obtain an R, G or B values could be seriously flawed – made worse when considering the non-linearity of RGB space in terms of visual perception.

Finally, as stated above, there are crucial differences between the visual perceptions of humans and non-human animals. The perception of a given colour signal to a human may be markedly different from that of the animal towards which the signal is directed. The fact that colour charts have numerous errors associated with them, especially self-made charts, in terms of human judgement, only emphasises the inadequacy of the method when used with respect to non-human animals. The fact remains that other animals' perceptions of a signal may drastically differ from our own.

## Conclusion

The inadequacy of colour charts as a means to estimate the colour of animal signals is not a new topic, yet too often researchers outside of the technical colour sciences have adopted this procedure, despite the serious implications of doing so. Theoretically, the use of colour charts is poor practice when considering signals aimed at non-human animals, since these will often have significantly different visual perceptions. Also, some colour spaces on which colour charts are based are not linear in the perceptual differences between one point in space and another, even for humans. This is the case for the RGB/HTML colour space used to create charts by Berggren & Merilä [1]. Even charts that are uniform in colour space are far from perfect and an active area of research in the human vision sciences. Our results cast further doubt on the use of charts to estimate colour, in that individual people vary in their colour matching choices, and that the light environment also can affect colour perception, albeit to a far smaller degree than printer variation. Matching between different people will be variable and error prone and, even if the experiments are all performed by the same individual, their perceptions can also change based on the environmental conditions. In the case of 'self-produced' printed colour charts, different charts vary in their properties, and the same printers may not produce equal steps between colour blocks on a chart, even if that is the case on a computer.

In some instances, access to expensive equipment such as spectrophotometers and calibrated digital cameras may be difficult. In this case, the use of a colour chart may be the only option, and is certainly better than an abstract description of an observed colour. However, we urge caution with the use of colour charts, and advocate that they are used only as a last resort. We would also not recommend that anyone produces self-made charts based on empirically or perceptually non-uniform colour spaces, and are extremely dubious of results obtained in this way. If colour charts are to be used, we recommend the use of a well studied, perceptually uniform colour space, such as CIELAB, with colour matching experiments undertaken by the same individual in as carefully controlled conditions as is possible.

## Methods

Whilst theoretical arguments indicate why the use of colour charts, in particular those based on RGB/HTML colour space, are a poor method to estimate the chromatic components of animal signals, we wished also to provide quantitative evidence. Our experiments aimed to show that there may be at least three problems with using humans to match the colour of an object to a set of charts, even discounting differences between human vision and that of other species. Firstly, perception of colour may vary between individuals. Secondly, the exact reproduction of a colour chart will vary depending on the printer from which the charts are produced (not to mention the toner levels and paper type). Thirdly, the light environment may also affect colour matching results.

### Colour Matching Experiments

We designed colour charts in Jasc Paint Shop Pro^® ^based on RGB values, consisting of coloured rectangles 68 mm by 20 mm in size, with 12 different rectangles per sheet, inserted into a Microsoft Word^® ^file for easy printing. Colour charts were of two types, red or blue-green, with RGB values ranging from 0,0,0 to 250,0,0 for the red charts, and 0,0,0 to 0,250,250 for the blue-green charts. Copies of each chart type were printed from eight different printer types. The quality of the printers ranged from relatively inexpensive office ink jet types, to high quality laser printers used by the University of Bristol Print Services (HP Colour LaserJet 2500, HP InkJet Combi, HP DeskJet 1220c, HP DeskJet 6127, Epson Stylus Photo 915, Epson Stylus Colour 760, Cannon LaserJet 2100, Cannon LaserJet 5100).

The experiment was a repeated-measures design, with each of the 11 subjects (normally sighted according to self-report) asked to match a colour sample to what they perceived to be the most similar colour on each of the printed colour charts, under each of four different lighting conditions. The order of testing was randomised across subjects, with the authors blind to which colour match was optimal, and the subjects blind to the experimental aims. For each chart and lighting condition, a different colour sample was selected at random from an envelope. In fact, to simplify the subsequent analysis, within the two categories of colour stimuli (red and blue-green), all the samples to be matched were nominally identical, but subjects were unaware of this. The samples were obtained from paint charts (Dulux, Slough, UK, 'Spring 04 colour card') and their similarity verified using spectrophotometry (see below). The pretence of random selection from an apparently large set of samples was introduced to discount the possibility that subjects would recognise the same card, and bias their choices to the same match with each choice made.

Colour matching experiments were performed under four different light conditions: a 150 Watt Xenon arc lamp (Light Support, Berkshire, UK), a 20 Watt desktop incandescent lamp (Philips, PL-Electronic-T), general laboratory fluorescent lighting (Sylvania T5 FHE, Raunheim, Germany), and outside skylight. Incandescent lights contain filaments heated to high temperatures, and typically emit light richer in longer wavelengths (giving a reddish tinge) [30]. The xenon arc lamp tends to have an output richer in short wavelengths. The outside conditions used to test people were under sunshine and cloud, but avoiding direct sunlight, and would tend to be white-ish [41]. The order that each colour chart (from the different printers) was presented, and the order of light conditions under which the charts were viewed, was randomised for each subject so that the possibility of any biases developed towards specific colour patches were controlled for.

### Spectrophotometry of Colour Charts

We aimed to quantify the properties of each colour rectangle on each of the colour charts from different printers via spectrophotometry. These results would also show how much variation exists between the different printed charts, which, in theory, should all show the same reflectance spectra. Reflectance measurements of each colour block on each chart was undertaken with a Zeiss MCS 230 diode array photometer, with illumination by a Zeiss CLX 111 Xenon lamp (Carl Zeiss Group, Jena) held at 45° to normal to reduce specular reflection. Measurements were taken normal to the surface, from a 2 mm area, recorded in 1-nm intervals from 300 to 700 nm, and expressed relative to a Spectralon 99% white reflectance standard (Labsphere, Congleton). White standard measurements were taken between measurements of each colour chart, to avoid error associated with drift in the light source and sensor. In all, 10 measurements, in different locations, were taken of each of the 12 colour blocks, on the 8 red and the 8 blue-green charts. Plus, 10 measurements were taken from a random sample of 8 red and 8 blue-green paint cards, giving a total of 2080 reflectance spectra measurements. For each colour block measured, or for the paint cards, the repeated samples were used to produce average spectra.

## Authors' contributions

ICC devised the idea of the paper and the general experiments. MS designed the colour charts, and undertook the colour matching experiments. ICC and MS undertook the spectrophotometry measurements. MS and ICC analysed the colour matching, and MS the spectophotometry results. MS & ICC wrote the manuscript.
